# The Willow Microbiome Is Influenced by Soil Petroleum-Hydrocarbon Concentration with Plant Compartment-Specific Effects

**DOI:** 10.3389/fmicb.2016.01363

**Published:** 2016-09-08

**Authors:** Stacie Tardif, Étienne Yergeau, Julien Tremblay, Pierre Legendre, Lyle G. Whyte, Charles W. Greer

**Affiliations:** ^1^Department of Natural Resource Sciences, McGill UniversitySainte-Anne-de-Bellevue, QC, Canada; ^2^Section of Microbial Ecology and Biotechnology, Department of Plant and Environmental Sciences, University of CopenhagenCopenhagen, Denmark; ^3^Energy, Mining, and Environment, National Research Council CanadaMontréal, QC, Canada; ^4^Centre INRS-Institut Armand-Frappier, Institut national de la recherche scientifiqueLaval, QC, Canada; ^5^Département de Sciences Biologiques, Université de MontréalMontréal, QC, Canada

**Keywords:** contamination, endophytes, microbiome, phytoremediation, rhizosphere, willow

## Abstract

The interaction between plants and microorganisms, which is the driving force behind the decontamination of petroleum hydrocarbon (PHC) contamination in phytoremediation technology, is poorly understood. Here, we aimed at characterizing the variations between plant compartments in the microbiome of two willow cultivars growing in contaminated soils. A field experiment was set-up at a former petrochemical plant in Canada and after two growing seasons, bulk soil, rhizosphere soil, roots, and stems samples of two willow cultivars (*Salix purpurea* cv. FishCreek, and *Salix miyabeana* cv. SX67) growing at three PHC contamination concentrations were taken. DNA was extracted and bacterial 16S rRNA gene and fungal internal transcribed spacer (ITS) regions were amplified and sequenced using an Ion Torrent Personal Genome Machine (PGM). Following multivariate statistical analyses, the level of PHC-contamination appeared as the primary factor influencing the willow microbiome with compartment-specific effects, with significant differences between the responses of bacterial, and fungal communities. Increasing PHC contamination levels resulted in shifts in the microbiome composition, favoring putative hydrocarbon degraders, and microorganisms previously reported as associated with plant health. These shifts were less drastic in the rhizosphere, root, and stem tissues as compared to bulk soil, probably because the willows provided a more controlled environment, and thus, protected microbial communities against increasing contamination levels. Insights from this study will help to devise optimal plant microbiomes for increasing the efficiency of phytoremediation technology.

## Introduction

Phytoremediation exploits the relationships between plants and their associated microbial communities to remediate contaminated environments. This cost- and energy-efficient technology offers a promising solution to the problem of worldwide soil pollution. The lengthy remediation rates associated with this technology, however, have severely impeded its capacity to compete on the market and have frequently been linked to the insufficient establishment of plant and microbial biomass (Huang et al., 2004; Sun et al., 2011) and/or the selection of suboptimal plant microbiomes (Siciliano et al., 2003; Bell et al., 2014a,b).

The plant microbiome is comprised of archaeal, bacterial, and fungal communities that are associated with the host in the rhizosphere (soil directly attached to the plant-root interface), phyllosphere (aboveground parts of plants), and endosphere (interior tissue of plants; Kowalchuk et al., 2010; Schlaeppi and Bulgarelli, 2014). The breakdown of organic pollutants such as petroleum hydrocarbons (PHCs), including polycyclic aromatic hydrocarbons (PAHs), in phytoremediation technology is driven by the interaction between the plants, and microorganisms (El Amrani et al., 2015). The stimulated microorganisms use organic contaminants as carbon and/or energy sources and in the process, partially, or completely breakdown these compounds into less toxic or less available substrates in the environment (Reichenauer and Germida, 2008). These processes occur within the plant itself but more commonly within the rhizosphere. The rhizosphere provides a favorable physical and chemical environment in which microorganisms strive resulting in increased microbial biomass and activity (Günther et al., 1996). This environment often boosts higher nutrient concentrations than the surrounding bulk soil due to the release of plant exudates, which are comprised of an array of different organic compounds including amino acids, flavonoids, aliphatic acids, organic acids, proteins, and fatty acids (Berg et al., 2005; el Zahar Haichar et al., 2008). Many of these plant secondary metabolites are structurally similar to organic contaminants (Singer et al., 2003) and as a result, the expression of hydrocarbon degradation genes is generally elevated in the rhizosphere (Yergeau et al., 2014), which increases degradation (Reichenauer and Germida, 2008). Several studies have shown that organic compounds such as PHCs (Yateem et al., 1999) and PAHs (Pradhan et al., 1998) are degraded more rapidly by rhizosphere communities than by surrounding bulk soil communities.

Endophytic microorganisms colonize the endosphere without harming their plant host. These microorganisms have been shown to metabolize pollutants and to impact plant fitness (Taghavi et al., 2005). Endophytes also often have plant growth promoting capacity, through nitrogen fixation (Doty et al., 2009), enhancement of phosphate availability (Verma et al., 2001), siderophore production (Rungin et al., 2012), phytohormones synthesis (Tanimoto, 2005), or decreasing ethylene levels (Glick et al., 1998).

Successful phytoremediation of PHC contaminated soils is highly dependent on the selection of an appropriate plant-microbiome system. Willows (*Salix* spp.) are fast growing, resilient plant species, which produce sizeable biomass, and create extensive root systems (Schnoor et al., 1995). They are genetically diverse with over 400 cultivar/species and can often be grown from cuttings (Newsholme, 2003; Pulford and Watson, 2003), an advantage for the efficient implementation of this technology in the field. *Salix* spp. have shown considerable promise in the phytoremediation of organic contaminants (Vervaeke et al., 2003). However, despite recent advances in this field, little is known about the intricate relationships formed between *Salix*, and its microbiome, knowledge essential to future plant microbiome engineering. Microbial phylotyping can provide critical information for the selection of optimized microbiomes which can lead to enhanced host performance traits such as survival, growth, and fitness (Mueller and Sachs, 2015; Yergeau et al., 2015). The central objective of this study is to understand how contamination affects the microbiome throughout the willow-microbiome holobiont and if this is similar between closely related species of willows. In order to attain this objective, bulk soil, rhizosphere soil, roots, and stems of *Salix purpurea* cv. Fish Creek and *Salix miyabeana* cv. SX67 growing in three PHC contamination concentrations were collected for DNA extraction, which was then amplified using bacterial 16S rRNA and fungal ITS genes-specific primers, and sequenced on a Ion Torrent Personal Genome Machine (PGM). Results from this study showed that the contamination concentration significantly influenced the willow microbiome, but not identically for all plant compartments. Fungi and bacteria also responded differently to contamination, willow species, and compartments.

## Materials and methods

### Experimental design and sampling

The site is located in Varennes, Québec, Canada (45°43N, 73°22W) at a closed petrochemical plant. This site was fully operational for 55 years, resulting in land contaminated with a large mixture of PHCs, including PAHs. It is divided into two main areas, one of which is non-contaminated (N1) with PHC concentrations found below the detection limit according to the Canada-wide standard for petroleum hydrocarbon (PHC) in soil (CWS, 2003) (< 100 mg/kg) in a preliminary survey performed in 2010 and a contaminated area. This study was conducted within the framework of a large phytoremediation pilot project and in addition to the control plot N1, 2 contaminated plots were selected for use, one of which had moderate contamination concentrations (C3), and one with high contamination concentrations (C5). Twelve soil samples from each plot were sent to Maxxam Analytics (Montréal, Québec, Canada) on the 9th of August, 2011 for F1-F4 hydrocarbon analysis, which represents the sum of aliphatic and aromatic hydrocarbon compounds with chain lengths of C6-C50. C3 had concentrations averaging 709 mg kg^−1^ [±339 (standard error)] while C5 had concentrations averaging 3590 mg kg^−1^ [±760 (standard error). Replicates (150) of 11 willow cultivars were planted in each plot in June, 2011 in a randomized block design for more details on the experimental design as well as physico-chemical analysis of soil see Bell et al. (2014a)]. Other factors such as soil pH, texture, moisture content, conductivity, and nutrient concentrations could have co-varied with contamination levels and have an effect on the plant microbiome but the very large differences in contamination levels as well as the proximity and similarity of the soils of the different plots likely resulted in the contamination being the main driver.

Sampling of all 3 plots at random for 2 cultivars (*Salix purpurea* “Fish Creek” and *Salix miyabeana* “SX67”) in triplicates was performed in November 2012, after two full growing seasons (average annual temperature of 5.3°C and average total yearly precipitation of 1018 mm). These cultivars were chosen because of their high yield field performance as well as their dissimilar backgrounds, Fish Creek being of European origin and SX67 being an Asian variety. To characterize the willow microbiome, 4 distinct compartments were targeted: bulk soil (~200 g of composite top 10 cm soil), rhizosphere soil (~200 g of composite root-adhering soil, top 10 cm soil), root tissue (~10 g), and stem tissue (~10 g). A total of 72 samples were collected (3 replicates × 2 cultivar × 3 contamination levels × 4 compartments).

### Surface sterilization of plant tissue and DNA extraction

Surface sterilization of root and stem tissue samples was first performed in order to cleanse the surface of the plant of any attached microorganisms, as these are not part of the endophytic flora (Escobar, 2012). Samples were rinsed with distilled water to wash off the remaining attached soil, and under aseptic conditions, were immersed in the following washes: 1 min in 100% ethanol; 1 min in 2.5% NaOCl; 10 min in 2.5% NaOCl under gentle shaking; 1 min in 100% ethanol; 30 s in autoclaved distilled H_2_O (this step was repeated three times). From the final H_2_O wash, 1 ml was plated on YTS_250_ to verify root surface sterility. Following this, tissue was macerated with a sterile pestle and mortar on a bed of dry ice until a fine powder was produced. DNA extractions on 250 mg of ground plant tissue samples or 250 mg of soil samples were performed using the MoBio PowerSoil® DNA extraction kit (MoBio, Carlsbad, CA, USA). Samples were eluted in 50 uL of autoclaved MilliQ H_2_O.

### PCR amplification and next-generation sequencing

For 16S rRNA gene amplification, soil DNA was amplified by PCR using the primers UnivBactF 9 (5′-GAGTTTGATYMTGGCTC-3′) and BSR534/18 (5′- ATTACCGCGGCTGCTGGC-3′), which amplified the V1-V2 hypervariable regions, as described in Yergeau et al. (2012). Plant tissue DNA was amplified by PCR, targeting the 16S rRNA gene using the primers 520F (5′-AGCAGCCGCGGTAAT-3′) and 799R2 (5′- CAGGGTATCTAATCCTGTT-3′) targeting the V2-V3 hypervariable regions and excluding the chloroplast 16S rRNA gene, as described in Edwards et al. (2007). To account for use of different primers and for downstream comparison of samples, a set of samples which were amplified with both primer sets were compared (see supplementary material). A co-inertia analysis was performed, comparing both datasets generated from different primers, and revealed a strong correlation between these datasets and in similar bacterial communities. Hence, we determined that downstream analyses comparing datasets generated with these different primer sets were valid. For the ITS region, soil and plant tissue DNA were amplified using the ITS1F (5′-CTTGGTCATTTAGAGGAAGTAA-3′) and ITS2 (5′-GCTGCGTTCTTCATCGATGC-3′) primer set as in Ghannoum et al. (2010). To distinguish between samples, unique multiplex identifier (MID) tags from the extended MID set recommended by Roche (2009) were integrated in the primers, as described in Sanschagrin and Yergeau (2014).

PCR reactions were performed in 20 μL volumes containing 12.5 μL HotStart Taq *Plus* Master mix (Qiagen, Germantown, MD, USA), 0.5–2 μL of template DNA, 0.4 μL of bovine serum albumin from 20 mg/mL stock and 0.625 μL of each forward and reverse primer from 20.0 μM stocks. For the 16S rRNA primers, cycling conditions were as follows: 5 min at 95°C, 25 cycles of 30 s at 95°C, 30 s at 55°C, and 45 s at 72°C, and a final elongation step of 10 min at 72°C. Amplification of 520F /799R2 amplicons used identical cycling conditions but for 30 cycles and with a 58°C annealing temperature. The amplification of a plant tissue sample could only be achieved when raising the number of cycles to 35. To evaluate the biases that this might cause, another sample that was previously amplified with 30 cycles was also amplified with 35 cycles and sequenced. The ordination coordinates and the community composition of the two datasets were highly similar (see supplementary material), suggesting that cycle number would have a negligible influence in downstream analyses. For the ITS primers, cycling conditions were as follows: 5 min at 95°C, 30 cycles of 60 s at 94°C, 60 s at 45°C, and 60 s at 72°C, and a final elongation step of 10 min at 72°C.

Amplicons were verified on 2% agarose gels and then gel purified using the PureLink® Quick Gel Extraction Kit (Invitrogen, Life Technologies, Carlsbad, CA, USA) and quantified using the Quant-iT PicoGreen dsDNA assay kit (Invitrogen, Life Technologies). Purified bacterial 16S rRNA gene amplicons were pooled in equimolar ratios separately for soil and endophyte samples, resulting in two pools of 36 samples. The same procedure was followed for the fungal ITS region amplicons, resulting in a grand total of four pools. Each pool was then sequenced following Sanschagrin and Yergeau (2014). Briefly, emulsion PCR was carried out with the Ion One Touch 200 template kit (Life Technologies, Carlsbad, CA) using the OneTouch ES instrument (Life Technologies). The resulting libraries were sequenced on the Ion Torrent PGM system (Life Technologies) using 314 chips with the Ion Sequencing 200 kit.

### Sequence data treatment

Sequences were analyzed using the procedure described in Tremblay et al. (2015). Since the bacterial primers used for endophytic and soil communities targeted different regions, they were processed separately as the alignments generated during the procedure would have only overlapped over ~15 bp. Soil and endophyte OTU tables were merged for further taxonomic classification and subsequent downstream analyses. OTU tables were rarefied to 1000 reads for bacterial datasets and 306 reads for ITS fungal datasets.

### Statistical analyses

All statistical analyses were performed in R v.2.15.2 (R Foundation for Statistical Computing; available at http://www.R-project.org). The effect of compartment, contamination and cultivar type on the bacterial and fungal community composition was tested. First, the square roots of UniFrac matrices were used for principal coordinate analyses (PCoA), which were carried out using the “pcoa” function of the “ape” package (Paradis et al., 2004). This unconstrained analysis was used in order to explore the patterns of bacterial and fungal OTU composition across samples. Following this, a distance-based redundancy analyses (dbRDA; Legendre and Anderson, 1999a) with the “rda” function and the “anova.cca” of the “vegan” package (Oksanen et al., 2008) was carried out in order to test the significance of these descriptors by multivariate statistical hypothesis testing. This statistical analysis was used to test the significance of the individual descriptors (plant compartment, contamination level, and cultivar type) on multispecies response variables (bacterial/fungal communities) in a multifactorial analysis-of-variance model as described in Legendre and Anderson (1999b). A partial dbRDA was used for testing the effect of compartment in order to control for the identity of the trees. The reported adjusted redundancy statistics R^2^ were obtained using the “RsquareAdj” function of the “vegan” package. The relative abundance of specific classified OTUs was represented by samples in a heatmap using the “pheatmap” function in the “pheatmap” package. Venn diagrams were created using the “venn.diagram,” “draw.triple.venn,” and “draw.quad.venn” functions in the “VennDiagram” package to visualize the number of OTUs that were shared between compartments, contamination, and cultivar type. Venn diagrams of soil and endophyte bacterial samples were produced separately because unique OTUs were formed for each dataset upon clustering.

Co-inertia analysis (Dolédec and Chessel, 1994) was used to determine whether bacterial and fungal communities were similarly affected by contamination and cultivar type. The OTU abundance data was first normalized with a hellinger transformation with the “decostand” function of the “vegan” package. Co-inertia analyses were then performed separately for each compartment with the “dudi.pca” and “coinertia” functions in the “ADE4” package (Dray and Dufour, 2007). The significance was tested by permutation using the “randtest” function in the “ADE4” package. Similarly, co-inertia analysis was used to determine the covariance between rhizosphere microbial communities and endophyte root and stem microbial communities.

The bacterial and fungal alpha diversities in soil and inside plant tissues were compared using a two-way ANOVA test with the “aov” function. When the bacterial soil data failed to meet the assumptions of parametric ANOVA after log, square root, and several power transformations, the non-parametric Kruskal-Wallis one-way test of variance was performed with the “kruskal.test” of the “pgirmess” package. A multiple comparison test, *post hoc* was then performed between treatments using “kruskalmc” functions of the “pgirmess” package in order to distinguish which treatments were significantly different from one another. An indicator species analysis was performed based on the IndVal index to identify microbial OTUs that were significantly correlated to high contamination using the “multipatt” function in the “labdsv” package.

## Results

### Bacterial community composition

**Figure 1:**
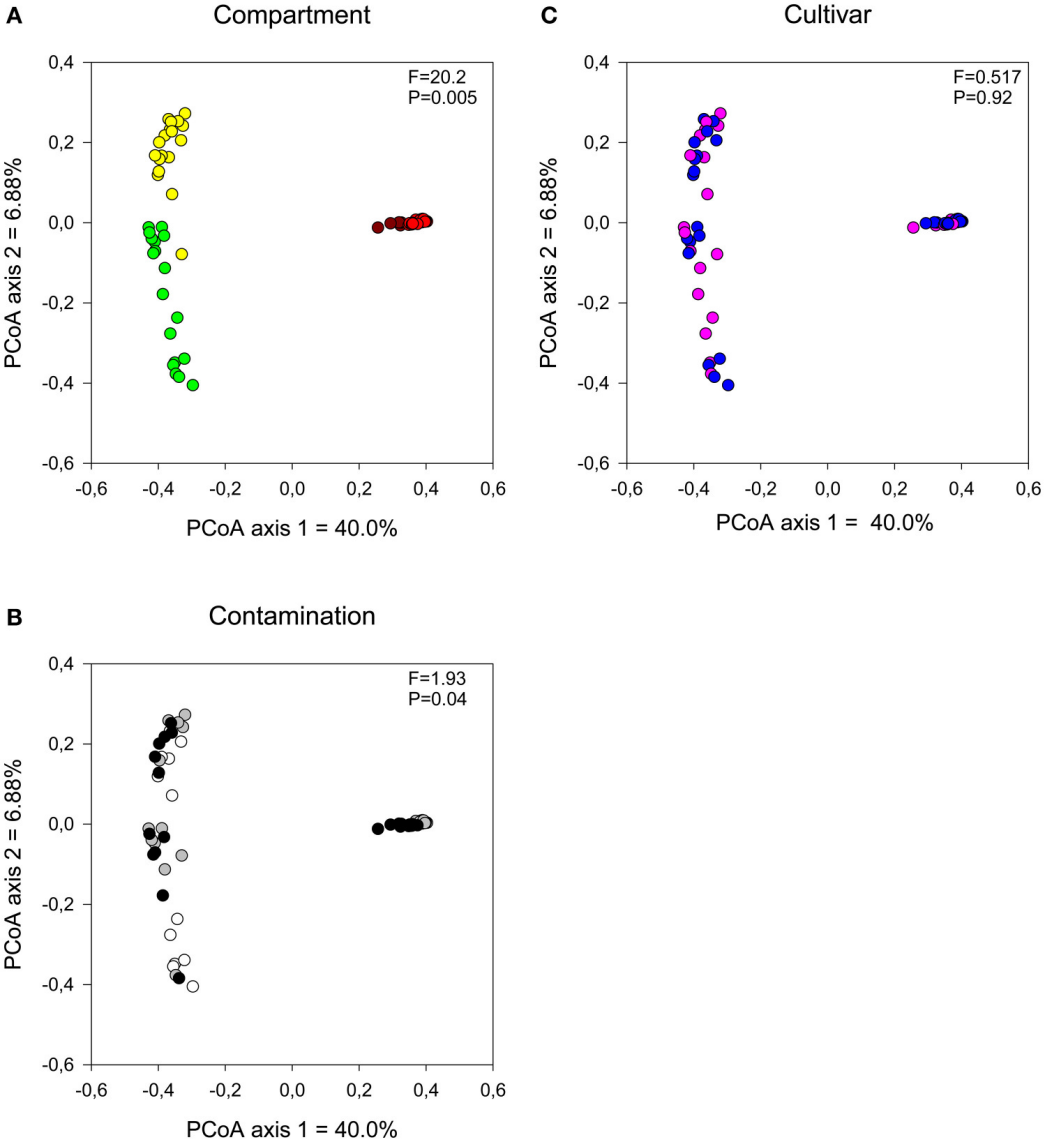
**Comparison of bacterial communities by (A) compartment, (B) contamination level, and (C) cultivar type**. Influence on soil and endophyte communities shown using principal component analysis based on UniFrac distance measures. *F*-ratio and *P*-values are from distance-based redundancy analyses (Compartment (controlling for the identity of the trees): brown, bulk; red, rhizosphere; yellow, root, and green, stem. Contamination: white, N1; gray, C3, and black, C5. Cultivar: pink, fish, and blue, SX67).

The influence of plant compartment, cultivar, and contamination levels on bacterial community composition was visualized using a PCoA ordination and tested for significance using distance-based redundancy analyses. Bacterial communities were primarily influenced by plant compartment (Figure 1A; partial dbRDA: adjusted *R*^2^ = 0.442, *F* = 20.2, *P* = 0.005), with some minor, but significant effect of contamination levels (Figure 1B; adjusted *R*^2^ = 0.0255, *F* = 1.93, *P* = 0.0411). Cultivar had no significant effects on bacterial community structure (Figure 1C; adjusted *R*^2^ = −0.00684, *F* = 0.517, *P* = 0.92).

**Figure 2:**
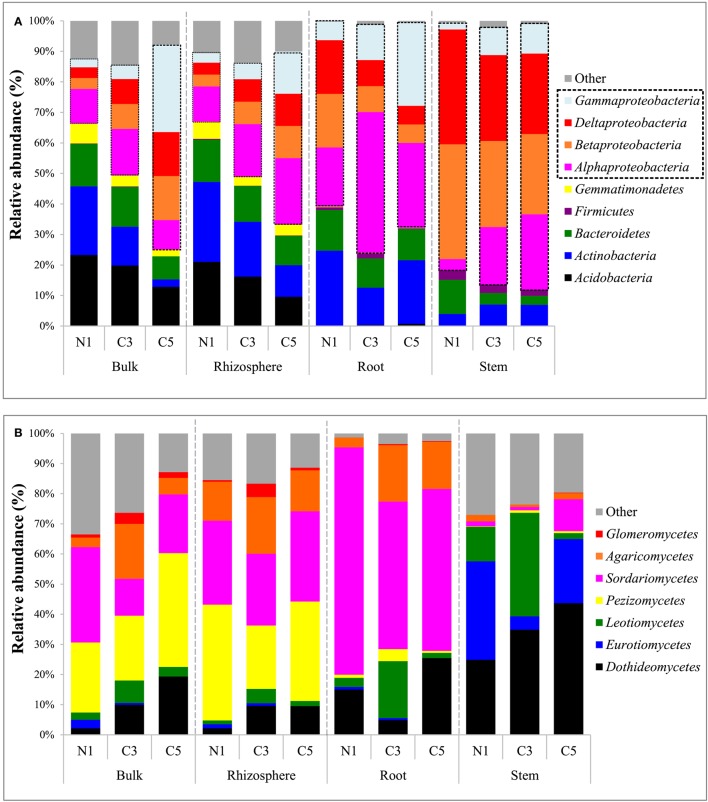
**(A)** Bacterial community composition of major phyla (and *Proteobacteria* classes) by averaged samples and **(B)** fungal community composition of major classes by averaged samples (N1, non-contaminated; C3, moderately contaminated; C5, high contamination).

**Figure 3:**
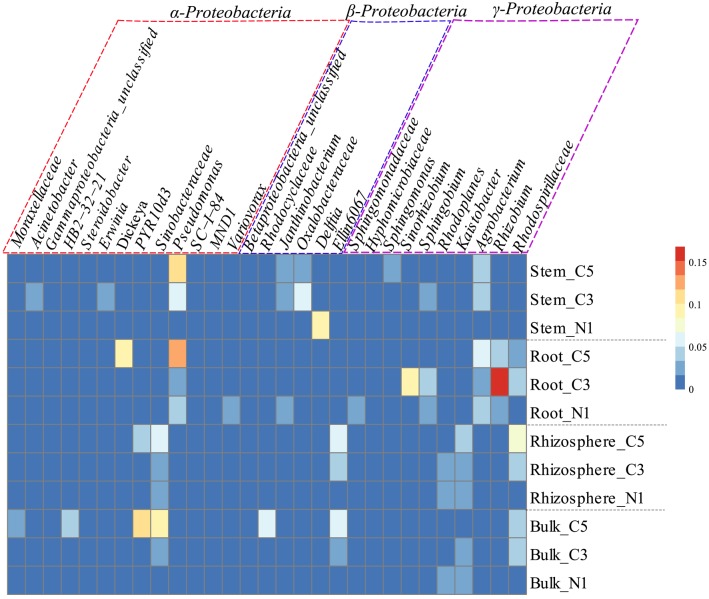
**Relative abundance (%) of the most dominant OTUs of the classes ***Alphaproteobacteria*** (purple box), ***Betaproteobacteria*** (blue box), and ***Gammaproteobacteria*** (red box), by averaged sample represented in a heatmap which was normalized by row, where cells go from blue to red as abundance increases**.

Bacterial community composition, both in the soil and plant, were primarily dominated by the *Proteobacteria* (Figure 2A). Soil communities were also colonized by members of the *Acidobacteria, Actinobacteria, Bacteroidetes, Chloroflexi, and Gemmatimonadetes* phyla. In plant tissues, the *Alphaproteobacteria* class, of the *Proteobacteria* phylum, dominated the root samples while the *Betaproteobacteria* class was predominant in the stem samples. Members of the *Actinobacteria, Bacteroidetes*, and *Firmicutes* phyla were also present but relatively not abundant inside plant tissues. The genus *Ralstonia*, part of the *Betaproteobacteria* class, dominated the stem samples, representing an average of 43% of classified genera for these samples, and was excluded from Figure 3 for visual representation purposes.

A striking increase in the relative abundance of *Proteobacteria* in soil communities was observed as the contamination increased, mainly due to an increase in the *Gammaproteobacteria* as well as *Alphaproteobacteria* classes (Figure 2A). This increase in the *Proteobacteria* is in contrast to the decreases observed in the *Actinobacteria* and *Acidobacteria* in soil samples. The increase in the *Gammaproteobacteria* in the roots and stems was related to increasing relative abundance of *Pseudomonas*, while it was related to members of the *Sinobacteraceae* family and the *PYR10d3* order in soils (Figure 3). In the root samples, the increase in the *Alphaproteobacteria* at medium contamination was related to an increased relative abundance of *Rhizobium, Sinorhizobium, Sphingobium*, and members of the *Rhodospirillaceae* (Figure 3). The genus *Agrobacterium* was relatively more abundant in the roots and stems at high contamination levels, while the members of the *Oxalobacteraceae* increased with contamination in the stem samples (Figure 3).

**Figure 4:**
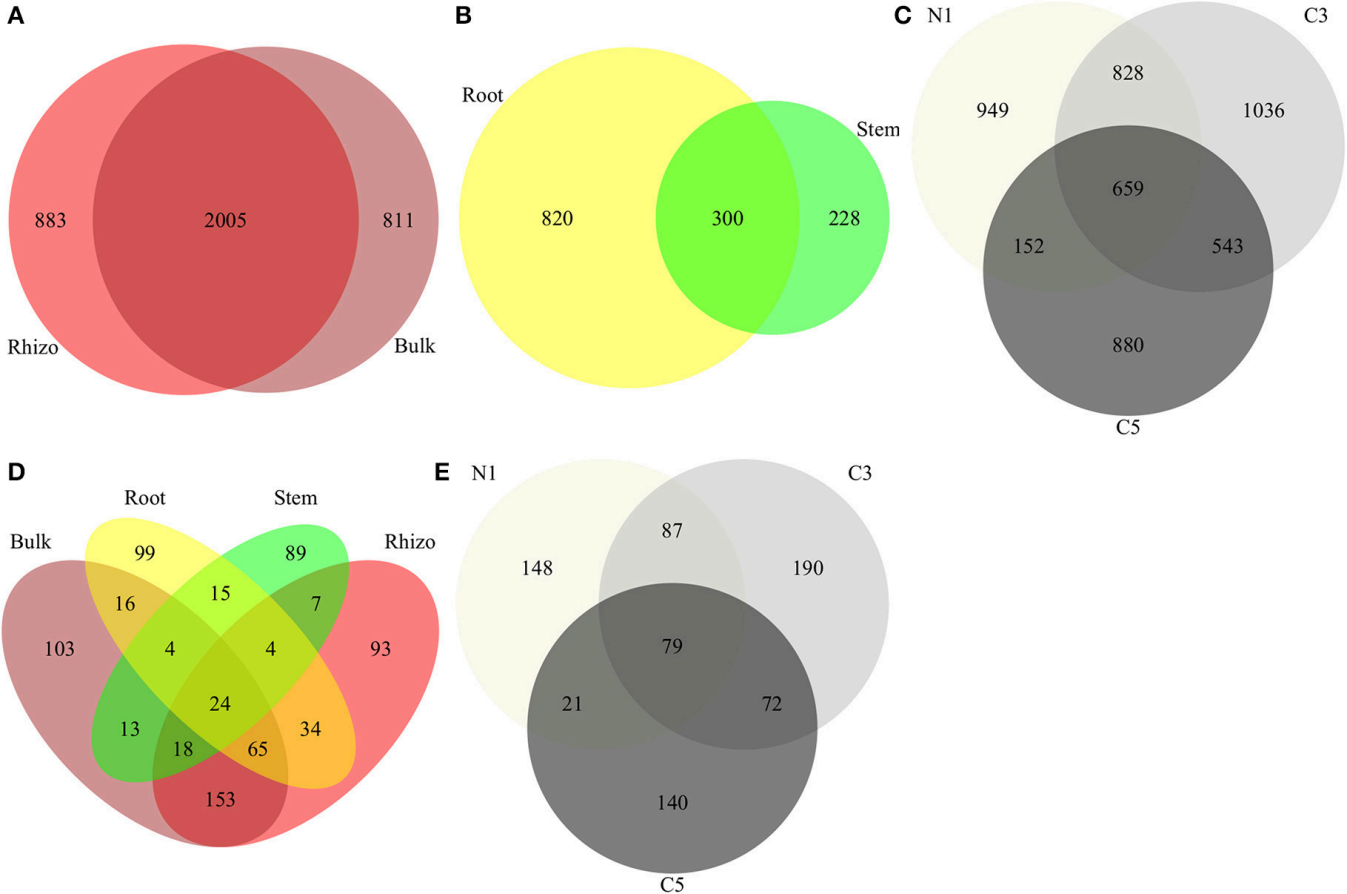
**Comparison of communities using Venn diagrams showing OTUs shared between (A) bacterial soil compartment, (B) bacterial endophyte compartments, and (C) bacterial OTUs by contamination level (D) fungal compartments and (E) fungal OTUs by contamination level (Contamination: N1, no contamination; C3, medium contamination; C5, high contamination)**.

In line with the tighter clustering of the bulk and rhizosphere soil samples as compared to the plant tissue samples in the PCoA ordinations (Figure 1), bulk and rhizosphere soil samples shared 54% of their OTUs (Figure 4A) while root and stem endophyte samples only shared 22% of their OTUs (Figure 4B). Samples from each contamination level harbored unique OTUs, with more shared OTUs between N1-C3 and C3-C5 than N1-C5 (Figure 4C).

### Fungal community composition

**Figure 5:**
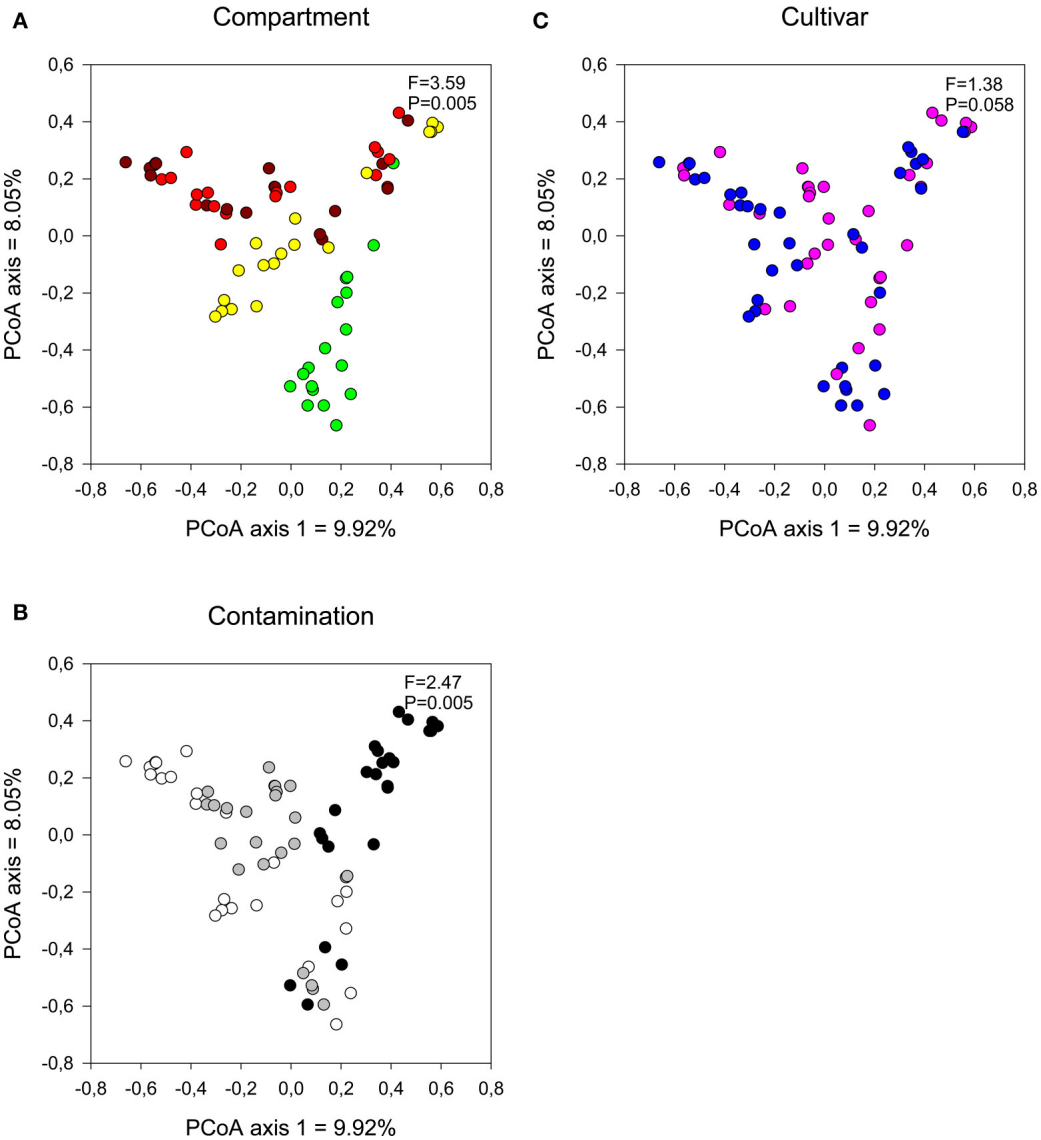
**Comparison of fungal communities by (A) compartment, (B) contamination level, and (C) cultivar type**. Influence on soil and endophyte communities shown using principal component analysis based on UniFrac distance measures with a distance-based redundancy analysis [Compartment (controlling for the identity of the trees): brown, bulk; red, rhizosphere; yellow, root, and green, stem. Contamination: white, N1; gray, C3 and black, C5. Cultivar: pink, fish, and blue- = SX67].

The contamination level had a more pronounced effect on the fungal communities than on the bacterial communities, as visualized by the clearer clustering of the C5 samples away from the N1 and C3 samples (Figures 5A,B) and the higher significance level in partial dbRDA tests (adjusted *R*^2^ = 0.0708, *F* = 3.70, *P* = 0.005). Plant compartment also significantly influenced the fungal community structure (Figure 5A; adjusted *R*^2^ = 0.0963, *F* = 3.59, *P* = 0.005), while the cultivar type did not have a significant effect on fungal communities (Figure 5C; adjusted *R*^2^ = 0.00534, *F* = 1.38, *P* = 0.0585).

**Figure 6:**
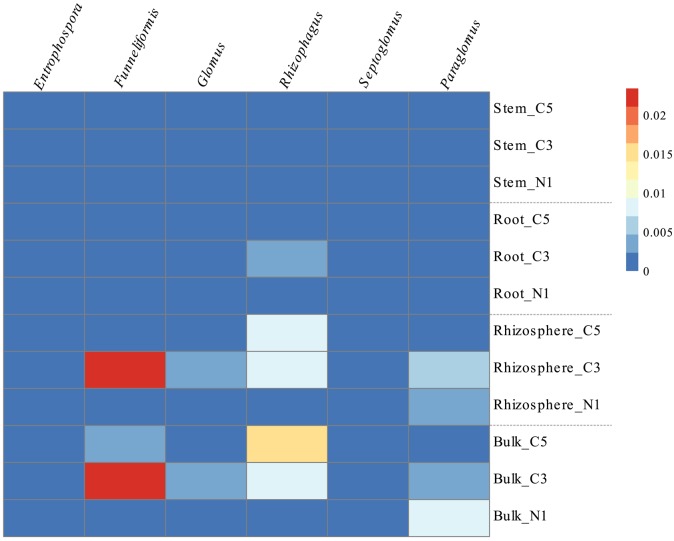
**Relative abundance (%) of the genera comprising the ***Glomeromycetes*** class by averaged sample represented in a heatmap, where cells go from blue to red as abundance increases**.

The soil fungal communities were dominated by the *Pezizomycetes* and *Sordariomycetes* with minor contributions from the *Dothideomycetes, Agaricomycetes*, and *Glomeromycetes*. The root fungal communities were primarily composed of fungi from the *Sordariomycetes* while the stem fungal communities were dominated by the *Dothideomycetes, Eurotiomycetes*, and *Leomycetes* (Figure 2B). Increasing contamination was associated with increased presence of the *Dothideomycetes* and *Pezizomycetes* in soils (Figure 2B). The *Agaricomycetes* were relatively more abundant in the intermediate contamination soils (C3; Figure 2B). The C3 soils also showed relatively higher abundance of *Glomeromycetes*, which is the class containing the arbuscular mycorrhizal fungi (AMF; Figure 2B). The genus *Funneliformis* of the *Glomeromycetes* showed large increases in relative abundance in the C3 soils (Figure 6). The AMF genera *Glomus, Rhizophagus Septoglomus* and *Paraglomus*, were also relatively more abundant in the C3 soils, with stronger effects in the rhizosphere as compared with the bulk soil (Figure 6). The AMF genus *Rhizophagus* increased its relative abundance with increasing contamination levels both in the soil and in the plant tissues (Figure 6).

Each compartment selected for unique fungal OTUs, with larger overlaps between bulk and rhizosphere soils but also between root and soil samples (Figure 4D). Similar to bacteria, contamination level selected for unique OTUs with N1 and C5 sharing considerably fewer OTUs than N1-C3 and C3-C5 (Figure 4E).

### Co-inertia analyses

Co-inertia analysis was used to test whether bacterial and fungal communities were being similarly influenced by contamination and cultivar type. Co-inertia analyses showed that bulk (*RV* = 0.961, *P* = 0.001) and rhizosphere (*RV* = 0.952, *P* = 0.001) bacterial and fungal communities were similarly influenced by these factors while root (*RV* = 0.814, *P* = 0.573) and stem (*RV* = 0.763, *P* = 0.593) communities were not. Similarly, co-inertia analyses was used to determine if the bacterial and fungal communities responded similarly to contamination levels across the different plant compartments. The RV coefficients for the co-inertia analyses were significant when comparing bacterial rhizosphere and root communities (*RV* = 0.913, *P* = 0.001) and bacterial rhizosphere and stem communities (*RV* = 0.874, *P* = 0.017). For the fungi, significant co-inertia coefficients were only found when comparing fungal rhizosphere and root communities (*RV* = 0.919, *P* = 0.001).

### Shannon diversity

For bacteria, soil and plant tissue samples were tested separately. For soil samples, Shannon bacterial diversity was significantly influenced by contamination (Kruskal-Wallis, Chi-squared = 21.5, *P* = 0.000021), with significant differences between N1 and C3 (*post hoc, Z* = 1.33, *P* = 0.05; Table 1). Shannon bacterial diversity in plant tissue samples was significantly influenced by compartment (two-way ANOVA, *F* = 14.6, *P* = 0.00082) and by the interaction term compartment^*^contamination (*F* = 3.70, *P* = 0.0399). Shannon fungal diversity was significantly influenced by compartment (two-way ANOVA, *F* = 26.5, *P* = 2.97 × 10^−10^), by contamination (*F* = 3.36, *P* = 0.0431), by cultivar (*F* = 4.85, *P* = 0.0324) as well as by the interaction term compartment^*^ contamination (two-way ANOVA, *F* = 2.59, *P* = 0.0298).

**Table 1:** **Shannon diversity of compartment, contamination, and cultivar type of bacterial and fungal communities**.

		**Bacteria**	**Fungi**
Compartment	Bulk	6.8 ± 0.63	4.2 ± 0.79
	Rhizosphere	7.0 ± 0.24	4.3 ± 0.77
	Root	4.8 ± 1.3	2.6 ± 1.0
	Stem	3.3 ± 1.2	2.6 ± 0.85
Contamination	N1	5.4 ± 2.2	3.4 ± 1.2
	C3	5.7 ± 1.7	3.8 ± 1.4
	C5	5.3 ± 1.5	3.2 ± 0.92
Cultivar	Fish	5.5 ± 1.7	3.2 ± 1.2
	SX67	5.4 ± 1.9	3.8 ± 1.1

*Contamination: N1, no contamination; C3, medium contamination; C5, high contamination*.

*Values are average ± standard deviation (compartment: N = 18; contamination: N = 24; cultivar type: N, 36)*.

### Indicator “species” analysis

The indicator species analysis was carried out on OTUs and the taxonomy at the genera level is given for the top 10 indicator OTUs for C3, C5, and C3+C5 plots in Table 2. The full list of indicator OTUs for contamination is provided for bacteria and fungi in Supplementary Tables 1 and 2, respectively. Many of the indicator OTUs could not be classified at the genus level. However, several of these OTUs matched the same genus or family, with some genera such as the ones associated with the *Cytophagaceae* family comprising 16 of the indicator OTUs associated with C5 contamination. Other important contributors in the C3 plot were *iii1–15* (9 OTUs), *Cytophagaceae* (7 OTUs), and *Sordariomycetes* (5 OTUs). In the C5*, PYR10d3* (15 OTUs)*, Rhodospirillaceae* (13 OTUs), *Sinobacteraceae* (6 OTUs), *DS-18* (6 OTUs), *Rhodocyclaceae* (6 OTUs), and *Zopfiella* (10 OTUs) played a significant role. Finally, *Cytophagaceae* (4 OTUs), *Rhodospirillaceae* (4 OTUs), *Geobacter* (3 OTUs), and *Sinobacteraceae* (3 OTUs) were present both in C3 and C5 plots in high numbers.

**Table 2:** **Top ten results of significant bacterial and fungal indicator species analysis of C3, C5, and combined C3:C5 contamination listed at the highest universally known taxonomy level**.

	**IndVal**	***P*-value**	**Rank**	**Taxonomy**	**IndVal**	***P*-value**	**Rank**	**Taxonomy**	**IndVal**	***P*-value**	**Rank**	**Taxonomy**
			**C3**				**C5**				**C3 + C5**	
	0.683	0.001	Family	*Rhodospirillaceae*	0.701	0.001	Family	*Alteromonadaceae*	0.677	0.001	Family	*Sinobacteraceae*
Bacteria	0.645	0.001	Class	*Deltaproteobacteria*	0.701	0.001	Phylum	*Gemmatimonadetes*	0.66	0.001	Class	*Betaproteobacteria*
	0.639	0.001	Genus	*Inquilinus*	0.677	0.001	Class	*Gammaproteobacteria*	0.634	0.003	Class	*Betaproteobacteria*
	0.62	0.009	Phylum	*Chloroflexi*	0.674	0.001	Class	*Gammaproteobacteria*	0.62	0.001	Order	*Rhizobiales*
	0.614	0.028	Class	*Betaproteobacteria*	0.672	0.001	Phylum	*Gemmatimonadetes*	0.615	0.046	Genus	*Novosphingobium*
	0.612	0.001	Class	*Acidobacteria*	0.671	0.001	Class	*Betaproteobacteria*	0.612	0.001	Genus	*Hyphomicrobium*
	0.598	0.003	Family	*Rhodospirillaceae*	0.67	0.001	Family	*Cytophagaceae*	0.612	0.002	Family	*Cytophagaceae*
	0.594	0.001	Class	*Betaproteobacteria*	0.669	0.001	Phylum	*Acidobacteria*	0.61	0.005	Genus	*Steroidobacter*
	0.584	0.001	Phylum	*Acidobacteria*	0.654	0.001	Class	*Gammaproteobacteria*	0.596	0.002	Family	*Streptomycetaceae*
	0.584	0.001	Phylum	*Acidobacteria*	0.645	0.001	Family	*Rhodospirillaceae*	0.58	0.032	Family	*Rhodospirillaceae*
Fungi	0.673	0.001	Family	*Lasiosphaeriaceae*	0.938	0.001	Genus	*Zopfiella*	0.721	0.023	Genus	*Alternaria*
	0.647	0.001	Genus	*Funneliformis*	0.828	0.001	Kingdom	*Fungi*	0.673	0.003	Family	*Thelephoraceae*
	0.645	0.001	Genus	*Phaeosphaeriopsis*	0.825	0.001	Genus	*Epicoccum*	0.661	0.002	Genus	*Geopora*
	0.607	0.001	Phylum	*Ascomycota*	0.807	0.001	Genus	*Zopfiella*	0.66	0.042	Genus	*Cladosporium*
	0.603	0.002	Phylum	*Ascomycota*	0.791	0.001	Genus	*Zopfiella*	0.598	0.032	Genus	*Alternaria*
	0.602	0.001	Order	*Helotiales*	0.786	0.001	Genus	*Sphaerosporella*	0.559	0.006	Genus	*Tomentella*
	0.592	0.001	Genus	*Phaeoseptoria*	0.761	0.001	Genus	*Zopfiella*	0.54	0.006	Genus	*Rhizophagus*
	0.577	0.001	Genus	*Mycoarthris*	0.758	0.001	Genus	*Leptosphaeria*	0.54	0.003	Phylum	*Ascomycota*
	0.577	0.001	Genus	*Apodus*	0.742	0.001	Phylum	*Ascomycota*	0.5	0.018	Phylum	*Ascomycota*
	0.542	0.004	Family	*Pyronemataceae*	0.709	0.001	Genus	*Zopfiella*	0.433	0.044	Family	*Thelephoraceae*

*Contamination: N1, no contamination; C3, medium contamination; C5, high contamination*.

## Discussion

The microbiome of *Salix* species growing in PHC-contaminated soil was significantly influenced by contamination with distinct variations amongst compartments and bacterial and fungal community profiles. The effect of contamination influenced not only the rhizosphere soil communities, but also plant tissue microbiomes. The effect of contamination on the bacterial and fungal community composition was less prominent in the rhizosphere and in the plant tissues as compared with the bulk soil, suggesting that the plant environment may be acting as a protective buffer zone for the microbial communities in the soil. For instance the rhizosphere may buffer the effect of contamination on communities because of increased nutrient availability (Berg et al., 2005; el Zahar Haichar et al., 2008; Badri et al., 2009) and increased expression of hydrocarbon degradation genes (Yergeau et al., 2014). The plant tissues are partly buffered against the adverse effects of contamination by providing a physical barrier against the majority of toxic compounds. It has been shown in previous studies, however, that low-molecular weight, or partially degraded organic contaminants can migrate within the plant and directly influence and shape the plant microbiome (Taghavi et al., 2005; Germaine et al., 2009). In this study we also found that plant tissue microbiomes were composed of some OTUs that were shared with the soil microbiomes. These results suggest that some rhizosphere microorganisms might colonize the endosphere and as such, the effect of contamination in the rhizosphere may be indirectly affecting the endophyte communities. Since these communities interact closely with the host plants, it provides a route for indirect effects of contaminants on plants. Conversely, the stress response of plants to contaminants might partly shape the endophyte communities by modifying the plant environment. An increased understanding of these interactions in the context of contaminated soil could help increase plant tolerance to contaminants resulting in a larger ecological range for plants or better growth and activity under high contamination.

Co-inertia analyses indicated that the bacterial and fungal communities of soil co-varied similarly with varying contamination levels, while plant tissue communities did not. This suggests that bacteria and fungi inhabiting roots and stems do not respond similarly to varying environmental conditions inside plant tissues, probably because of differences in growth patterns, metabolism, or colonization ability. In fact, with changing contamination levels, root, and stem bacterial communities responded similarly to rhizosphere bacterial communities, while the fungal stem communities did not, indicating a larger gap between soil fungi and fungi colonizing plant aerial parts, than for bacteria. Wearn et al. (2012) studied the endophyte communities associated with grassland forbs and found a remarkable difference between the fungal communities found in the root and shoots, suggesting a lack of systemic growth from one tissue to another. Our study suggests that although root tissues were susceptible to colonization by soil fungi, perhaps stem tissues may be more resilient to colonization from the soil.

In contrast to the general decrease in bacterial diversity with increasing contamination levels, soil fungal diversity appeared to be maximal at moderate contamination. This increased diversity corresponded with an increased relative abundance of the arbuscular mycorrhizal fungi (*Glomeromycetes*). AMF form symbiotic relationships with plants, directly promoting plant growth by capturing nutrients such as phosphorus, sulfur, and nitrogen (Schmidt et al., 2010). AMF increased in relative abundance in the C3 and for some genera, in the C5 plot, as compared with N1, both for soil communities and some root communities. This increase is seen very clearly with the genus *Rhizophagus*, that may have the potential to degrade hydrocarbons (Calonne et al., 2014). We also observed a marked increase in the *Pezizomycetes* with contamination, a fungal class that was shown to have a strong association specifically with North American willow cultivars growing in highly PHC-contaminated soils, mainly related to the species *Sphaerosporella brunnea*, an ectomycorrhizal fungi (EMF; Bell et al., 2014a). Within the endophyte communities, *Dothideomycetes*, which contains members that are able to degrade hydrocarbons (Bell et al., 2014a) and have been associated with plant health (Popp et al., 2006), also increased with contamination. In line with these findings, the indicator fungal species analysis of contamination identified four important mycorrhizal fungi, *Rhizophagus, Funneliformis, Sphaerosporella*, and *Geopora. Rhizophagus*, and *Funneliformis*, are well known AMF species associated with plant growth promotion (Wu et al., 2014; Padmavathi et al., 2015). Species of *Geopora*, been found to be the principal EMF colonists of willows planted for restoration in fly ash and have been hypothesized to survive well under harsh environmental conditions (Gehring et al., 2014). These identified genera may be playing an important role in contaminant degradation, plant health and plant growth promotion.

For bacteria, the *Proteobacteria*, particularly the *Gammaproteobacteria*, increased considerably with increasing contamination. These findings are consistent with many previous studies that have demonstrated the hydrocarbon degradation potential of many members of this class (Arun et al., 2008; Sopeña et al., 2013). Notably, the family *Sinobacteraceae* (Gutierrez et al., 2013) and the order *PYR10d3* (Singleton et al., 2006), which have been associated with hydrocarbon degradation in previous studies, increased with contamination. Indeed, *Sinobacteraceae* were also identified as indicator taxa for the highly contaminated samples. Of interest, the *Alphaproteobacteria* class increased with contamination selectively in the rhizosphere soil. The *Rhodospirillaceae* family, previously reported to increase its expression of the alkane 1-monooxygenase gene (alkB, C5-C16 alkane substrate) in the rhizosphere of willows (Pagé et al., 2015), was found here to be relatively more abundant in highly contaminated rhizosphere soil. The indicator species analysis of contamination also identified other interesting potential players from different classes of the *Proteobacteria* phylum including the *Rhodocyclaceae* family, which contains members that can degrade pyrene (Singleton et al., 2006) and phenanthrene (Singleton et al., 2005). *Geobacter* species are important anaerobic bacteria that have the ability to oxidize organic compounds such as hydrocarbons and halogenated compounds (Holmes et al., 2007). The anaerobic micro niches in the soil could accommodate these species and as they were recurrently associated with contamination, these microorganisms could play a role in anaerobic hydrocarbon degradation. Further studies aiming at isolating the bacteria and fungi identified here would be necessary to confirm their precise roles in phytoremediation and their potential for plant inoculation approaches.

Endophyte community shifts were also highly linked to the *Proteobacteria*. Notably, the genera *Pseudomonas, Dickeya*, and *Steroidobacter* of the *Gammaproteobacteria* class and *Sinorhizobium, Sphingobium*, and *Rhizobium* of the *Alphaproteobacteria* class increased their relative abundance in the roots with contamination. In addition to being part of the natural willow endophyte microbiome (Doty et al., 2009), previous studies have found that these classes contain many important plant-growth promoting organisms, harboring multiple plant-beneficial properties (Bruto et al., 2014). The indicator species analysis of contamination identified bacteria in the microbiome such as the *Cytophagaceae* (Xu et al., 2014) and *Rhodospirillaceae* (Madigan et al., 1984), which are known nitrogen fixers and may be associated with plant health. Interestingly, the *Streptomyces*, identified as significant indicators of C5, as well as both C3 and C5, has species that can promote fungal growth and mycorrhizal formation (Tokala et al., 2002; Tarkka et al., 2008). Members of this genus have also demonstrated plant growth promoting abilities by effectively helping plants mobilize and acquire nutrients, control plant pathogens and produce siderophores (Verma et al., 2011). In addition, this analysis also identified some genera that were previously associated with hydrocarbon degradation within the endophyte communities. Past studies have found that *Novosphingobium* has been linked with the degradation of aromatic compounds such as phenol, aniline, nitrobenzene, and phenanthrene (Liu et al., 2005) as well as other PAHs (Sohn et al., 2004). In addition, *Sphingomonadaceae* have been associated with hydrocarbon degradation (Liang and Lloyd-Jones, 2010) while *Steroidobacter* contains members that are steroidal hormone degrading bacteria (Fahrbach et al., 2008). The increased relative abundance of these microorganisms with increasing contamination in the plant microbiome could suggest that the plant host may be recruiting these organisms for survival in these highly toxic environments. Alternatively, this increase could also be explained by an increase in nutrient (i.e., hydrocarbon) availability within the plant tissues as a result of the translocation of contaminants within the plant tissues.

## Conclusions

This study has provided a unique view into the microbiome of willows growing in PHC-contaminated soils. We found that contamination was the primary factor structuring not only the rhizosphere, but also plant tissue microbiomes. Plant tissue microbiomes were composed of OTUs shared with the soil microbiomes, but also of unique OTUs. The presence of the plant provided a protective buffer zone against contamination in the rhizosphere and in the root and stem tissue, resulting in less drastic effects of increasing contamination on microbial community composition, as compared with the bulk soil. Species diversity generally decreased as contamination increased with the exception of increased fungal diversity at moderate contamination, which was linked with increased AMF relative abundance. In addition, increasing contamination resulted in shifts in the composition of the microbiome, favoring putative hydrocarbon degraders and microorganisms putatively associated with beneficial plant growth effects. The indicator species analysis identified several key microorganisms associated with contamination. Further isolation, characterization, and inoculation studies, however, will be essential to test out their ability to stimulate remediation processes. Our study has shown that contamination affects the microbiome of willows, but with differences between plant compartments and between bacteria and fungi. This information will prove to be important to devise and engineer optimal plant microbiomes for the efficient phytoremediation of organic contamination.

## Author contributions

ST designed and carried out this study, collected data, performed the analysis, and wrote the manuscript. EY contributed to design, data processing, statistical analyses, and preparation of the manuscript. JT participated in data processing and analysis. PL participated in data and statistical analyses. LW participated in design and manuscript revision. CG participated in the design, planning, data analysis, and manuscript revision.

## Funding

This work was supported by the Genome Canada and Genome Québec funded GenoRem Project.

### Conflict of interest statement

The authors declare that the research was conducted in the absence of any commercial or financial relationships that could be construed as a potential conflict of interest.
