# Supplementary material for: The Willow Microbiome Is Influenced by Soil Petroleum-Hydrocarbon Concentration with Plant Compartment-Specific Effects
Source: Front Microbiol. 2016 Sep 8;7:1363. doi: 10.3389/fmicb.2016.01363 (PMC5015464; doi:10.3389/fmicb.2016.01363)
Supplement: Supplementary file 3 [file DataSheet1.DOCX]

Supplementary Material

**The Willow Microbiome is Influenced by Soil Petroleum-Hydrocarbon Concentration with Plant Compartment-Specific Effects**

**Stacie Tardif^*^, Étienne Yergeau, Julien Tremblay, Pierre Legendre, Lyle G. Whyte and Charles W. Greer**

*** Correspondence:**

Stacie Tardif

[stta@plen.ku.dk](mailto:stta@plen.ku.dk)

# Supplementary Data

**Supplementary Material**

## 533R/343F primers vs. 799R2/520F endophyte primers: Primer Bias

Different sets of primers were used for the amplification of the bacterial 16S soil DNA and plant tissue DNA. Whereas the universal primers amplified one part of the 16S gene (V1-V2 region), the endophyte primers amplified another part (V2-V3 region). A statistical test was performed to determine whether a primer bias was introduced which would in fact render downstream comparison between these datasets problematic.

To test the primer effect on the amplified bacterial communities, we used two datasets, generated from the same samples. These datasets were produced using two different sets of primers. Dataset 1 included the OTUs generated from root and stem tissue using universal 16S primers. This dataset was heavily polluted with chloroplast sequences and after quality processing, had a small number of sequences (rarefied at 36 sequences/sample). Dataset 2 included sequencing results of root and stem tissue using endophyte specific primers (rarefied at 1000 sequences/sample).

A co-inertia analysis was performed to search for common structures between these two datasets. This analysis was chosen as it is a very flexible symmetrical canonical analysis which allows the use of data tables with identical number of rows, in this case sample number and with different number of columns, in this case OTU number. The abundance community composition data was transformed using the hellinger transformation of the ‘decostand’ function in R. The functions ‘dudi.pca’, ‘coinertia’ and ‘plot.coinertia’ of the ‘ADE4’ package were then used for the co-inertia analysis. This analysis reported a significant correlation between the two datasets (RV = 0.715, P = 0.004). From these results, we concluded that both primer sets were amplifying similar bacterial communities and would not impact downstream statistical analyses comparing datasets generated with these different primer sets.

## PCR: 30 vs. 35 cycles

The amplification of a plant tissue sample, 1FISH1.4, could only be achieved when raising the number of cycles from 30 to 35. To control for this deviation of protocol and to confirm that the additional cycles would not affect the results, we amplified sample 3SX674.3, which was previously amplified at 30 cycles, at 35 cycles. DNA sequencing was performed and results were compared using a phylogeny-based Unifrac PCoA (Supplementary Figure 1), carried out using the ‘pcoa’ function of the ‘ape’ package and ‘ggplot2’ of the ‘ggplot2’ package in R as well as a taxonomic graph representing the relative abundance of the ten most abundant classes (Supplementary Figure 2). The ordination showed that the microbial communities generated from both these samples were exceptionally similar, as demonstrated in the overlap of these samples on the plot. Furthermore, the taxonomic graph confirmed the similarity in amplified bacterial communities, as both these samples were nearly indistinguishable from one another. Indeed, Acinas et al. (2005) reported the absence of PCR-bias between two libraries constructed from PCR products amplified for different number of cycles. It was therefore concluded that sample 1FISH1.4 could be included in our dataset and used in downstream analyses.

**References**

Acinas, S. G., et al. (2005). "PCR-induced sequence artifacts and bias: insights from comparison of two 16S rRNA clone libraries constructed from the same sample." Applied and Environmental Microbiology **71**(12): 8966-8969.

# Supplementary Figures and Tables

## Supplementary Figures

Supplementary Figure 1: Comparison of bacterial communities by replicate and PCR cycles using principal component analysis based on UniFrac distance measures.

Supplementary Figure 2: Relative abundance of 10 most dominant classes by contamination level and replicates (Contamination: N1=non-contaminated, C3=moderately contaminated, C5=highly contaminated).
